# Subclinical Macular Edema Detected by Spectral-domain Optical Coherence Tomography (SD-OCT) in HLA-B27 Positive Anterior Uveitis

**Published:** 2014

**Authors:** Marilita M Moschos

**Affiliations:** Department of Ophthalmology, University of Athens, Greece

**Keywords:** HLA-B27, Macula, Optical Coherence Tomography, Uveitis

## Abstract

**Purpose: **To assess foveal thickness by spectral-domain optical coherence tomography (SD-OCT) during acute anterior uveitis (AAU) in HLA–B27 patients.

**Methods**: Foveal thickness was measured at baseline and after clinical resolution of the uveitis. Data of the affected eyes of 11 patients were compared to those of a fellow, healthy eyes and to those of counterpart volunteers.

**Results:** At baseline mean foveal thickness was 190 ± 28 µm in affected eyes, 166 ± 20 µm in fellow eyes (p < 0.001), and 162±14 µm in control group (p = 0.01). On the second OCT evaluation, no significant change was observed in affected eyes while the difference versus fellow eyes and control group remained statistically significant.

**Conclusions:** A significant increase in foveal thickness was observed in eyes with HLA –B27 AAU when compared with healthy eyes. The difference persisted for at least one month, despite full clinical and functional recovery.

## INTRODUCTION

About 50% of patients presenting with non-granulomatous acute anterior uveitis (AAU) are HLA-B27 antigen positive. This incidence, however, increases to over 70% in subjects affected by recurrent episodes of ocular inflammation (1). Acute anterior uveitis in HLA-B27 positive patients present as usual; with an acute onset, mild to severe ocular pain, red eye, photophobia, and a small decrease in visual acuity (VA) (2, 3). In HLA-B27 associated forms, men are twice more likely to be affected than women; the disease is most often unilateral with a greater risk of recurrence (1, 2). Posterior pole alterations occur commonly in susceptible patients after recurrent attacks or prolonged disease (4, 5). Anterior vitritis, true vitritis with white exudative debris in the region of the ora serrata accompanied with retinal vasculitis and phlebitis, is seen in 30% to 63% of HLA-B27 positive patients, while cystoid macular edema (CME) occurs in 10 - 30% compared to 8% in HLA-B27 negative subjects (4,6).

Optical coherence tomography (OCT) is a contemporary method of obtaining high-resolution cross-section images of the retina, allowing precise measurement of retinal thickness. It uses infrared light to detect relative changes in reflection at optical interfaces, based on the principle of low-coherence interferometry (7). Optical coherence tomography is a safe, non-invasive technique, highly suitable for repeated macular measurements in inflammatory ocular diseases (8). Currently, available OCT models reach a theoretical axial resolution of 10 µm, with a high degree of reproducibility in localizing and measuring macular thickness (9).

In order to investigate a possible increase in foveal thickness in cases of ocular inflammation with predominant anterior segment involvement, we prospectively evaluated a series of HLA-B27 positive individuals with unilateral AAU using OCT.

## MATERIALS AND METHODS

In this prospective, controlled study, patients were recruited between January 2012 and July 2013 from the Department of Ophthalmology of the University of Athens (Greece).

Inclusion criteria were the occurrence of unilateral AAU and the presence of HLA-B27 antigen (any subtype). Exclusion criteria were the presence of any opacities of the media (cornea, lens, aqueous and vitreous humor) sufficient to prevent fundus examination and/or OCT scanning, any previous ocular surgery (cataract, glaucoma, vitreoretinal), any ongoing topical treatment for glaucoma, the presence of macular hole and epiretinal membranes or the presence of age-related macular disease and diabetic retinopathy.

Eyes of affected patients were first examined by OCT 0 to five days after the onset of AAU; then 4±1 weeks later, with obvious clinical signs of healing. Clinical measurements were taken at least 5 days after discontinuation of therapy. Best corrected VA (Logarithm of the Minimum Angle of Resolution -- log MAR units) was recorded; intra ocular pressure (IOP) was measured in all patients, and they were all examined by the slit lamp. Gonioscopy and funduscopy (after dilatation with 1% tropicamide) were performed. Optical coherence tomography was performed simultaneously in both eyes while age-matched healthy volunteers were examined as an external control group. Treatment consisted of an hourly administration of topical 1% prednisolone, a cycloplegic agent (3% scopolamine or 1% atropine) twice a day and a steroid ointment at night. Therapy was progressively tapered every four to five days until complete resolution of the uveitis.

All patients underwent macular OCT scanning (Spectral-domain OCT (Spectralis SD-OCT, Heidelberg Engineering), carried out by an experienced hand (MM). To measure foveal thickness, images were obtained with a 5 mm horizontal and vertical scan line, analyzed by the software function “retinal thickness”. The caliper was positioned at the lowest point of the foveal profile, and the two measurements averaged for each patient. Finally, the results were compared using the paired and unpaired student “t” test. Consistency of localization and the scanning process, together with the coefficient of variation were thus assessed.

## RESULTS

Eleven patients (seven men and four women, mean age 40 ± 16 yrs.) presenting with unilateral AAU displaying a positive HLA-B27 antigen were included in the study. Fourteen healthy volunteers (seven men and seven women, mean age 39 ± 11 yrs., 22 eyes) established the control group.

The coefficients of variation were assessed in the two centers, measuring 5.3% (SD: 5 µm) and 6.1% (SD: 6 µm), respectively. Study group’s initial and final VA, as well as the OCT results of the affected and of the healthy fellow eyes are all detailed in Table I (data of the control group not shown).

Mean foveal thickness at the baseline were 190±28 µm in the affected and 166 ± 20 µm in the fellow eyes (p < 0.001), in addition to 162 ± 14 µm in the control group (p = 0.01). In the study group eyes, mean VA increased from 0.16 ± 0.06 log MAR, to 0.05 ± 0.18 log MAR at the last OCT examination. Acute anterior uveitis clinically healed in all patients after 26 ± 9 days from the beginning of treatment. One patient developed persistent CME despite the fact that anterior segment inflammation had resolved. This subject was excluded from the second examination data due to the continuation of local and systemic anti-inflammatory treatment. At the second OCT evaluation (4 ± 1 weeks after the diagnosis), results for mean foveal thickness in the study eyes revealed 179 ± 21 µm, with no significant difference in the baseline (p = 0.35). Conversely, the difference between this latter cohort when compared to both the healthy fellow eyes, and the control eyes remained statistically significant (p = 0.02 and 0.05, respectively).

**Table 1:** Visual Acuities and the Oct Assessment Results in the Study and in the Fellow Eyes

Patient	initial VA AE^1^ (logMAR)	final VA AE^1^ (logMAR)	1^st^ OCT AE^2^ (μm)	2^nd^ OCT AE^2^ (μm)	1^st^ OCT FE^3^ (μm)	2^nd^ OCT FE^3^ (μm)
1	0,24	0,00	212	169	183	145
2	0,18	0,00	208	164	175	144
3	0,12	0,00	201	191	185	181
4	0,08	-	236	-	201	-
5	0,12	-0,10	181	189	153	185
6	0,30	0,02	157	181	149	137
7	0,22	0,04	171	144	159	157
8	0,10	0,00	134	161	130	135
9	0,16	0,56	207	498	157	162
10	0,12	0,10	188	201	175	168
11	0,14	0,00	192	210	161	149

¹ Initial and final VA. AE = Affected Eye. FE = Fellow healthy Eye.

² OCT values in the affected eyes at the baseline and at the second evaluation.

³ OCT values in the fellow healthy eyes at the baseline and at the second evaluation.

## DISCUSSION

Changes in macular profile have been extensively documented in posterior uveitis. To detect early macular changes in AAU, we used OCT investigation of macular thickness in HLA-B27 related AAU. Mean foveal thickness of 11 eyes affected by uveitis was significantly increased in the inflamed eye during unilateral AAU when compared to the contralateral eye or to normal subjects (collected as external controls). A new OCT scan was performed in those same eyes after 4 ± 1 weeks. At this time, inflammation was clinically resolved in 10 eyes; VA improved, and treatment stopped for at least five days. One eye developed clinical CME, confirmed by angiography. This patient was excluded from the next round of the examination statistics as the steroid therapy could not be discontinued. At the second evaluation, mean foveal thickness decreased in both the study and the fellow eye groups even though the former still showed higher values (179 ± 21µm vs. 156 ± 18µm). Surprisingly, the change between the initial and final OCT evaluation was not significant in the study eyes. The difference between the affected and fellow eyes did, however, remain significant (p = 0.02). The coefficients of variation and standard deviation found in this study are very similar to earlier results where macular evaluation was performed by OCT (7, 8). Moreover, our patients had sufficient VA to allow good fixation, with no significant retinal alterations except in one case, thus allowing accurate localization of the fovea. Usually, CME occurs in 10-30% of HLA-B27 positive patients (4, 8). The presence of retinal edema is classically suspected in the presence of a decrease in VA. Nussenblatt et al. found a strong correlation between angiographic evidence of macular thickening in uveitis and VA (logMAR) (10). Whatsoever, Antcliff et al. were unable to observe this association using OCT (8). Macular edema results from diseases causing rupture of the inner and/or outer blood-retinal barrier. Edema may develop from flooding of the extracellular space by proteins and fluids or from insufficient depletion of albumin and other osmotically active molecules. Such molecules bind water to cause edema (11). Their intricate depletion might be due to the external limiting membrane and entanglements in extracellular traffic flow within the retina. Contrary to surgical trauma, which triggers a transient inflammatory response, endogenous uveitis produces a constant release of inflammatory mediators. The course of inflammatory macular edema is not; therefore, necessarily self-limited, and can develop and persist long after the onset of the inflammation (12). Data collected in this series show that a significant increase of foveal thickness appeared in eyes of patients with AAU when compared with the fellow, healthy eye. This difference persisted for at least one month, despite full clinical and functional resolution of the disease suggesting that some changes occur in the retinal osmotic equilibrium during typical anterior uveitis. Given the absence of any ophthalmoscopic sign and the complete recovery of VA, this macular edema should be considered subclinical. The increase in the extent of study group and the prolongation of follow up are mandatory to better define whether and when the correspondence between the two eyes can be restored.

## References

[1] Rothova A, van Veenedaal WG, Linssen A, Glasius E, Kijlstra A, de Jong PT (1987). Clinical features of acute anterior uveitis. Am J Ophthalmol.

[2] Mapstone R, Woodrow JC (1975). HL-A 27 and acute anterior uveitis. Br J Ophthalmol.

[3] Zervas J, Tsokos G, Papadakis G, Kabouklis E, Papadopoulos D (1977). HLA-B27 frequency in Greek patients with acute anterior uveitis. Br J Ophthalmol.

[4] Bayen H, Bayen MC, De Curzon HP, Espinasse-Berrod MA, Manderieux N, Furia M, Campinchi R (1988). Involvement of the posterior eye segment in HLA B27(+) iridocyclitis Incidence Value of surgical treatment. J Fr Ophtalmol.

[5] Rodriguez A, Akova YA, Pedroza-Seres M, Foster CS (1994). Posterior segment ocular manifestations in patients with HLA-B27-associated uveitis. Ophthalmology.

[6] Rosenbaum JT (1989). Characterization of uveitis associated with spondyloarthritis. J Rheumatol.

[7] Hee MR, Puliafito CA, Wong C, Duker JS, Reichel E, Rutledge B, Schuman JS, Swanson EA, Fujimoto JG (1995). Quantitative assessment of macular edema with optical coherence tomography. Archives of Ophthalmology.

[8] Antcliff RJ, Stanford MR, Chauhan DS, Graham EM, Spalton DJ, Shilling JS, Ffytche TJ, Marshall J (2000). Comparison between optical coherence tomography and fundus fluorescein angiography for the detection of cystoid macular edema in patients with uveitis. Ophthalmology.

[9] Goebel W, Kretzchmar-Gross T (2002). Retinal thickness in diabetic retinopathy: a study using optical coherence tomography (OCT). Retina.

[10] Nussenblatt RB, Kaufman SC, Palestine AG, Davis MD, Ferris FL 3rd (1987). Macular thickening and visual acuity Measurement in patients with cystoid macular edema. Ophthalmology.

[11] Marmor MF (1999). Mechanisms of fluid accumulation in retinal edema. Doc Ophthalmol.

[12] Guex-Crosier Y (1999). The pathogenesis and clinical presentation of macular edema in inflammatory diseases. Doc Ophthalmol.

